# Protective effects of oxymatrine against arsenic trioxide-induced liver injury

**DOI:** 10.18632/oncotarget.12478

**Published:** 2016-10-05

**Authors:** Li Li, Qinghai Liu, Long Fan, Wei Xiao, Lei Zhao, Yu Wang, Weiguang Ye, Fei Lan, Bin Jia, Hua Feng, Changman Zhou, Xiuqin Yue, Guogang Xing, Tianlong Wang

**Affiliations:** ^1^ Department of Anesthesiology, Xuanwu Hospital, Capital Medical University, Beijing, China; ^2^ Department of Anatomy & Histology, School of Basic Medical Sciences, Peking University Health Science Center, Beijing, China; ^3^ Department of Anesthesiology, the First Affiliated Hospital of Xinxiang Medical University, Henan, China; ^4^ Department of Neurobiology, School of Basic Medical Sciences, Peking University, Beijing, China

**Keywords:** oxymatrine, arsenic trioxide, liver, HO-1, Nrf-2

## Abstract

Oxymatrine, a quinolizidine natural drug extracted from *Sophora japonica*, has been reported to have neuroprotective effect and cardioprotective effect. However, the protective effect of oxymatrine on arsenic trioxide (As_2_O_3_)-induced liver injury has not been reported. In the present study, we investigated the protective effects of oxymatrine on As_2_O_3_-induced liver injury in rats. Male Wistar rats were administrated 3mg/kg As_2_O_3_ intravenous injection on alternate days for 4 days. Oxymatrine was given 1 h before As_2_O_3_ treatment. The results showed that oxymatrine inhibited As_2_O_3_-induced hepatic pathological damage, liver ROS level and MDA level in a dose-dependent manner. As_2_O_3_ decreased the antioxidant enzymes SOD, GPX, and CAT activity and the decrease was inhibited by treatment of oxymatrine. Furthermore, oxymatrine attenuated the retention of arsenic in liver tissues and improved the expression of Nrf2 and HO-1. In conclusion, our results suggested that oxymatrine protected against As_2_O_3_-induced oxidative damage by activating Nrf2/HO-1 signaling pathway.

## INTRODUCTION

Arsenic is a hazardous substance of global concern that is present in soil, drinking water, and food [1]. Arsenic often causes severe health hazards such as dermatosis, diabetes, and cancers, despite of its beneficial role in the treatment of acute promyelocytic leukemia (APL) [2, 3]. Studies showed that arsenic affects all organ systems of human and liver is one of the most important target organs for arsenic [4]. Previous reports demonstrated that arsenic-induced liver injury was closely associated with oxidative stress [5]. Arsenic exposure has been reported to produce reactive oxygen species and depress the antioxidant defense system which leading to the oxidative damage of liver tissues [6]. Recently, studies showed that antioxidants had therapeutic effects against arsenic-induced liver injury [7, 8]. Nrf2 is a redox-sensitive transcription factor that plays a critical role in cellular antioxidant defense [9]. Once stimulation, Nrf2 translocates to the nucleus to initiate transcription of cytoprotective genes such as HO-1 [10]. Studies showed that activation of Nrf2 could protect against arsenic trioxide-induced injury.

Oxymatrine, the major quinolizidine alkaloid isolated from the root of *Sophora flavescens Ait* (kushen), has been reported to have anti-inflammatory, anti-tumor, and antioxidant effects [11, 12]. Previous reports showed that oxymatrine exerted a protective effect on ischemia or ischemia/reperfusion damage in liver, intestine and heart [13, 14]. Oxymatrine has been reported to protect rat brains against permanent focal ischemia [15]. Oxymatrine also protected against experimental hepatic fibrosis [16]. In addition, oxymatrine was found to prevent adriamycin-induced cardiac injury in rabbits, which was associated with its antioxidant and anti-apoptotic activities [17]. However, the protective effect of oxymatrine against arsenic trioxide-induced liver injury has not been reported. Thus, in the present study, we aimed to investigate the protective effects of oxymatrine against arsenic trioxide-induced liver injury.

## RESULTS

### Oxymatrine reduced As_2_O_3_-induced liver histopathologic changes

**Figure 1:**
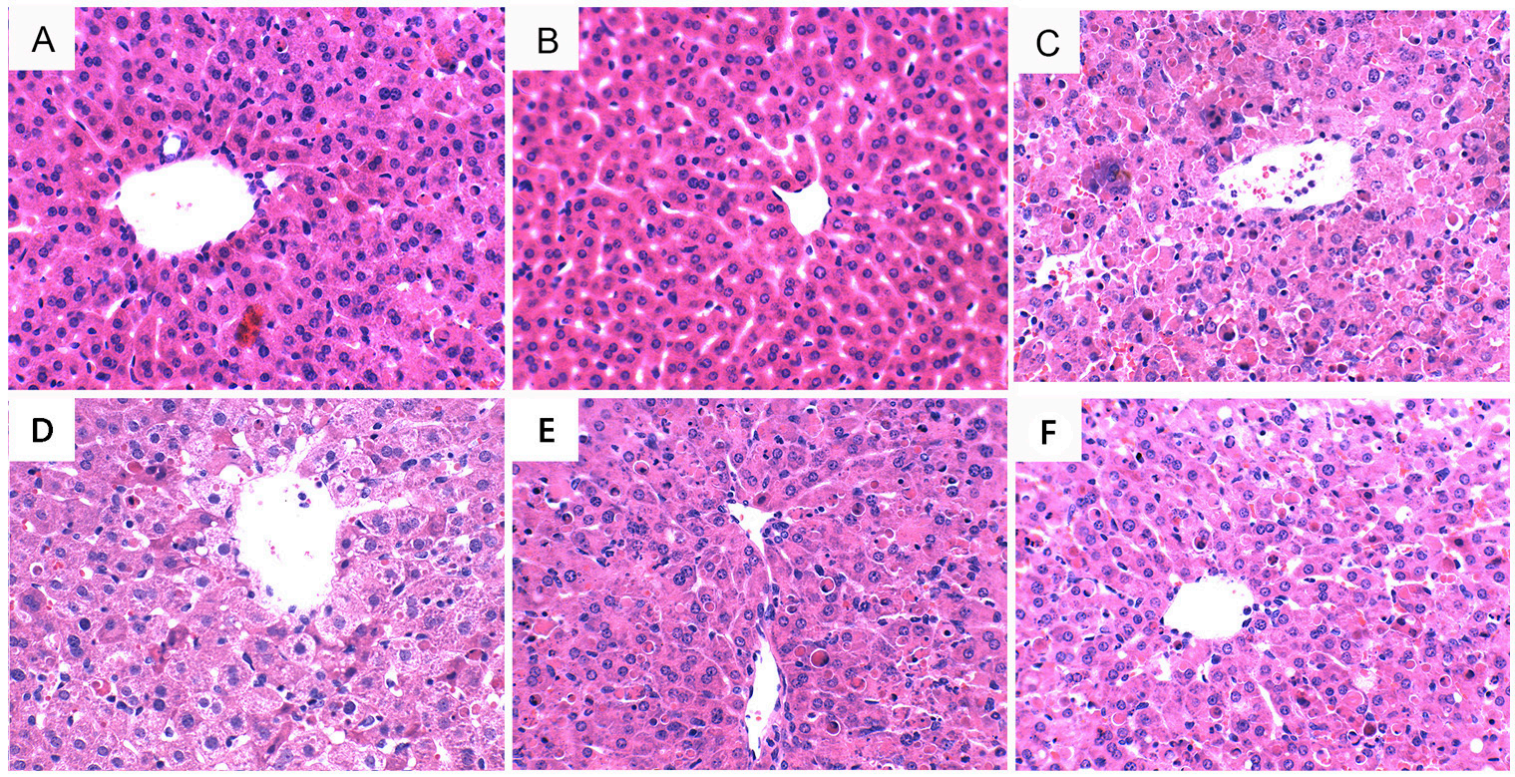
Effects of oxymatrine on As_2_O_3_-induced liver histopathologic changes Representative histological changes of liver obtained from mice of different groups. **A**. Control group, **B**. Oxymatrine (50mg/kg) group, **C**. As_2_O_3_ group, **D**. Oxymatrine + As_2_O_3_ (12.5 mg/kg) group, **E**. Oxymatrine + As_2_O_3_ (25 mg/kg) group, **F**. Oxymatrine + As_2_O_3_ (50 mg/kg) group (Hematoxylin and eosin staining, magnification 200×).

The effects of oxymatrine on As_2_O_3_-induced liver histopathologic changes were detected in this study. The results showed that liver tissues of control and oxymatrine-treated groups showed normal lobular architecture and cellular structure. Liver sections of As_2_O_3_-treated group showed significant pathologic changes, such as extensive areas of portal inflammation, inflammatory cell infiltration and cellular necrosis. However, the pathological changes of liver sections were attenuated in As_2_O_3_ + oxymatrine (12.5, 25, 50mg/kg) treated group (Figure 1).

### Effects of oxymatrine on As_2_O_3_-induced antioxidant enzymes SOD, GPX, and CAT activity

**Figure 2:**
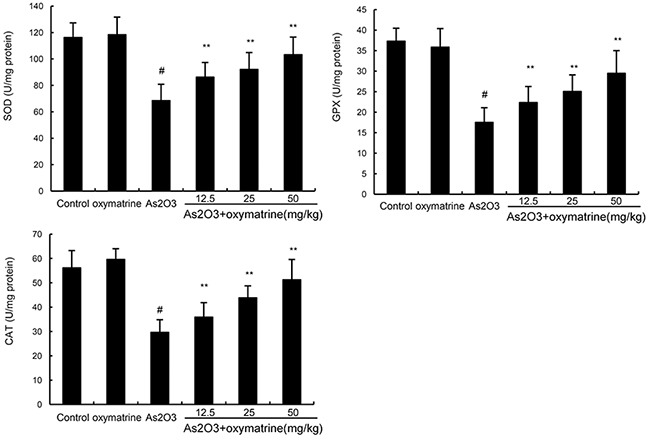
Effects of oxymatrine on As_2_O_3_-induced antioxidant enzymes SOD, GPX, and CAT activity The values presented are the mean ± SEM (n=12 in each group). p#<0.01 vs. control group, p*<0.05, p**<0.01 vs. As_2_O_3_ group.

The effects of oxymatrine on As_2_O_3_-induced antioxidant enzymes SOD, GPX, and CAT activity was shown in Figure 2. The results showed that compared to the control group and oxymatrine-treated group, the levels of SOD, GPX, and CAT decreased significantly in As_2_O_3_-treated group. However, the inhibition of SOD, GPX, and CAT levels by As_2_O_3_-was revised by treatment of oxymatrine (12.5, 25, 50mg/kg).

### Effects of oxymatrine on As_2_O_3_-induced ROS and MDA levels

**Figure 3:**
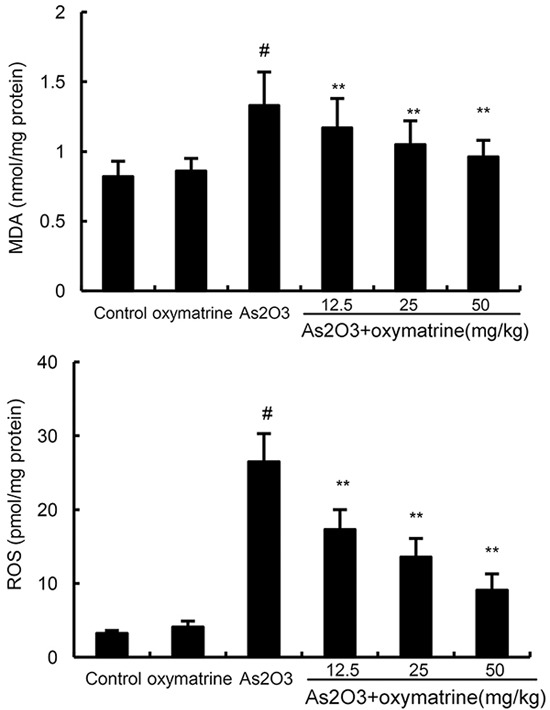
Effects of oxymatrine on As_2_O_3_-induced ROS and MDA levels The values presented are the means ± SEM (n=12 in each group). #p*<0.01 vs*. control group, *p*<0.05* and **p*<0.01 vs*. As_2_O_3_ group.

The effects of oxymatrine on As_2_O_3_-induced ROS and MDA levels were shown in Figure 3. The results showed that ROS and MDA levels increased significantly in As_2_O_3_-treated group in comparison with control group. However, the levels of ROS and MDA in As_2_O_3_ + oxymatrine (12.5, 25, 50mg/kg) treated group decreased significantly in comparison with As_2_O_3_-treated group.

### Effects of oxymatrine on As_2_O_3_-induced ALT and AST levels in serum

**Figure 4:**
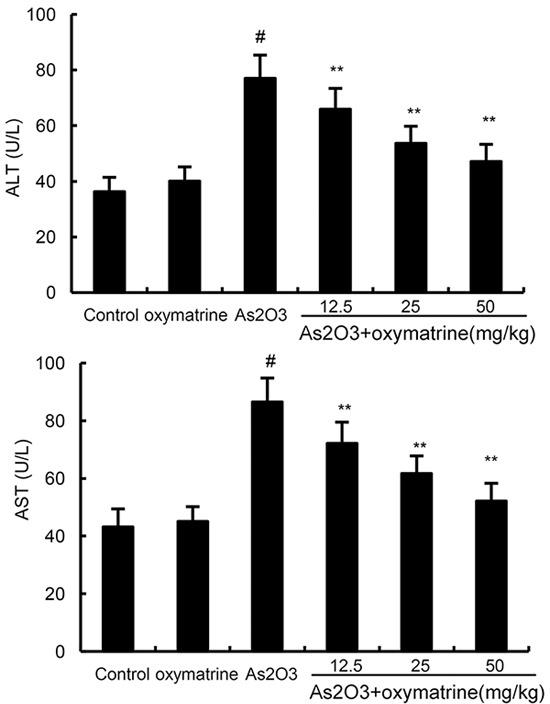
Effects of oxymatrine on As_2_O_3_-induced ALT and AST levels The values presented are the means ± SEM (n=12 in each group). #p*<0.01 vs*. control group, *p*<0.05* and **p*<0.01 vs*. As_2_O_3_ group.

The effects of oxymatrine on As_2_O_3_-induced ALT and AST levels were shown in Figure 4. As shown in Figure 4, the levels of ALT and AST of As_2_O_3_-treated group increased significantly than that of control group. However, the levels of ALT and AST in As_2_O_3_ + oxymatrine (12.5, 25, 50mg/kg) treated group decreased significantly in comparison with As_2_O_3_-treated group.

### Effects of oxymatrine on As accumulation in liver tissues

**Figure 5:**
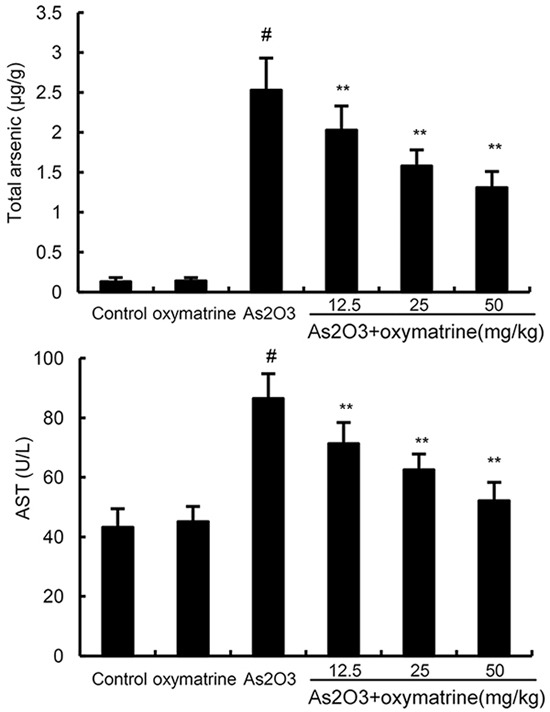
Effects of oxymatrine on As accumulation in liver tissues The values presented are the means ± SEM (n=12 in each group). #p*<0.01 vs*. control group, *p*<0.05* and **p*<0.01 vs*. As_2_O_3_ group.

The results showed that compared to the control group, As_2_O_3_ resulted in a significant increase in the arsenic concentration of liver tissues. However, treatment of oxymatrine (12.5, 25, 50mg/kg) significantly attenuated As_2_O_3_-induced accumulation of arsenic in liver tissues (Figure 5).

### Effects of oxymatrine on Nrf2 and HO-1 expression

**Figure 6:**
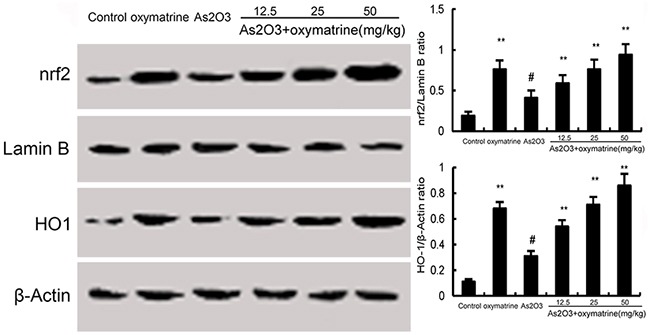
Effects of oxymatrine on Nrf2 and HO-1 expression The values presented are the means ± SEM (n=12 in each group). #p*<0.01 vs*. control group, *p*<0.05* and **p*<0.01 vs*. As_2_O_3_ group.

The effects of oxymatrine on Nrf2 and HO-1 expression were detected by Western blot analysis. As shown in Figure 6, As_2_O_3_ treatment increased the expression of Nrf2 and HO-1. However, oxymatrine (12.5, 25, 50mg/kg) up-regulated the expression of Nrf2 and HO-1 induced by As_2_O_3_.

## DISCUSSION

Oxymatrine, a quinolizidine natural drug extracted from *Sophora japonica*, has been reported to have antioxidant effect [12]. In the present study, we investigate the protective effects of oxymatrine on As_2_O_3_-induced toxicity in liver and oxidative stress in rats. The results showed that oxymatrine exhibited protective effects on As_2_O_3_-induced liver injury. Oxymatrine protected against As_2_O_3_-induced liver injury through activating Nrf2 signaling pathway.

Serum AST and ALT were used as biochemical indicator of liver injury [18, 19]. Our results showed that the levels of AST and ALT increased significantly in rats exposure to As_2_O_3_. Elevated levels of serum AST and ALT in As_2_O_3_-treated rats were associated with the extensive alterations in the pathological changes of liver tissues. Treatment of oxymatrine remarkably inhibited As_2_O_3_-induced AST and ALT production which suggesting oxymatrine could attenuate liver damage. Meanwhile, histological analysis demonstrated that oxymatrine attenuated liver pathologic changes, including inflammatory cell infiltration and cellular necrosis. These results indicated oxymatrine had protective effects against As_2_O_3_-induced liver injury.

Previous studies showed that As_2_O_3_-induced liver injury is associated with increased oxidative stress in liver tissues [20]. Arsenic exposure could induce the production of ROS which played critical roles in arsenic-induced toxicity [21, 22]. Lipid peroxidation generates a variety of relatively stable decomposition end-products, such as MDA, which can be measured as indirect indicators of oxidative stress [23]. In this study, we found that the levels of ROS and MDA in liver tissues increased after As_2_O_3_ exposure. Treatment of oxymatrine significantly inhibited As_2_O_3_-induced ROS and MDA production. Arsenic-induced damage in the antioxidant system involves several mechanisms such as altered SOD, CAT and GPX expression [24]. In this study, our results showed that the inhibition of SOD, GPX, and CAT levels by As_2_O_3_-was abolished by treatment of oxymatrine. These results indicated that oxymatrine exhibited antioxidant effects in As_2_O_3_-induced liver injury. Nrf-2 has been reported to play important roles in the induction of antioxidant enzymes against oxidative stress [25, 26]. HO-1 expression is controlled by the transcription factor Nrf-2 [27]. Recent studies showed that Nrf-2 signaling pathway played a protective role in As_2_O_3_-induced liver injury [28]. Our results showed that the increases in Nrf2 and HO-1 expression were augmented by oxymatrine.

In conclusion, the present study demonstrated that oxymatrine attenuated As_2_O_3_-induced oxidative damage in liver tissues by activating Nrf2/HO-1 signaling pathway. Oxymatrine may be an agent for preventing and treating As_2_O_3_-induced liver injury.

## MATERIALS AND METHODS

### Reagents

Oxymatrine (purity>98%) was purchased from Beijing SL Pharmaceutical Co., Ltd. (Beijing, China). As_2_O_3_ parenteral solution (10mg/ml) was purchased from Harbin Yida Pharmaceutical Company Ltd. (Harbin, China). GPX, SOD, CAT, and MDA determination kits were provided by the Jiancheng Bioengineering Institute of Nanjing (Nanjing, Jiangsu, China). Antibodies specific for Nrf2, HO-1, Lamin B, and β-actin were purchased from Cell Signaling Technology Inc (Beverly, MA). All other chemicals were of reagent grade.

### Animals

Seventy-two healthy male Wistar rats (110-130 g) were purchased from the Center of Experimental Animals of Shandong University (Shangdong, China). The rats were housed under standard conditions (temperature, 23±2°C; humidity: 60±5%). The rats were acclimatized to the environment for 6 days prior to the experiments. The rats had free access to water and food. All animal experiments carried out in this study were approved by the NIH Guide for the Care and Use of Laboratory Animals.

### Experimental protocol

A total of 72 rats were randomly divided into six groups (n=12 each group): normal control group, oxymatrine (50mg/kg) treatment group, As_2_O_3_ exposure group, and As_2_O_3_ + oxymatrine (12.5, 25, 50mg/kg) treatment group. In control group, rats were given equal amount of 0.9% normal saline. In oxymatrine treatment group, rats were given by an intraperitoneal injection of oxymatrine (50mg/kg). In the As_2_O_3_ exposure group, rats were administrated 3mg/kg As_2_O_3_ intravenous injection on alternate days for 4 days. In As_2_O_3_ + oxymatrine treatment group, rats were given by an intraperitoneal injection of oxymatrine (12.5, 25, 50mg/kg) 1 h before As_2_O_3_ administered. On the 8th day, rats were killed and the blood samples and livers from each group were collected for various biochemical analyses.

### Histological analysis

The liver tissues were collected and fixed in 10% formalin. The liver tissues were dehydrated, embedded in paraffin, and sliced at 4μM thickness. Then, the sections were stained with hematoxylin and eosin (H&E) reagent and visualized with a microscope (Olympus, Japan).

### Analysis of oxidative stress and antioxidant defense

Liver MDA and ROS level, the antioxidant enzymes SOD, GPX, and CAT activity were detected by using commercial kits purchased from the Jiancheng Bioengineering Institute of Nanjing (Nanjing, Jiangsu, China).

### Blood clinical analyses

Blood samples were collected and centrifuged at 3000 g for 8 min to obtain serum. The ALT and AST levels were measured using test kits purchased from the Jiancheng Bioengineering Institute of Nanjing (Nanjing, Jiangsu, China).

### Determination of total arsenic in the liver

The liver tissue was digested in HNO_3_-HCLO_4_ solution for 48 h at 130°C. Then the digested samples were diluted with deionized water. The concentrations of arsenic were detected using atomic fluorescence spectrometry.

### Western blot analysis

Proteins of liver tissues were extracted using T-PER Tissue Protein Extraction Reagent Kit according to the manufacturer's instructions (Thermo). The protein concentration was determined through BCA method. 40 μg proteins were separated on 10% SDS-PAGE gel and transferred onto PVDF membranes. After blocking with 5% skim milk for 2 h, the membranes were incubated with the specific primary antibodies Nrf-2 (1: 1000), HO-1 (1: 1000) at 4 °C overnight. After washing three times, the membranes were probed with HRP-conjugated secondary antibody at room temperature for 2h. Blots were then developed with the ECL Plus Western Blotting Detection System (Amersham Life Science, UK).

### Statistical analysis

The results are expressed as the mean ± SEM of three independent experiments. For comparison among groups were determined by one-way ANOVA followed by the Tukey post-hoc test. The P< 0.05 was considered statistically significant.
